# Harms to ‘others’ from alcohol consumption in the minimum unit pricing policy debate: a qualitative content analysis of UK newspapers (2005–12)

**DOI:** 10.1111/add.12427

**Published:** 2014-01-08

**Authors:** Karen Wood, Chris Patterson, Srinivasa Vittal Katikireddi, Shona Hilton

**Affiliations:** MRC/CSO Social and Public Health Sciences Unit, University of GlasgowGlasgow, UK

**Keywords:** Alcohol, alcohol policy, content analysis, harms to others, media, qualitative research

## Abstract

**Background and aims:**

Minimum unit pricing is a fiscal intervention intended to tackle the social and health harms from alcohol to individual drinkers and wider society. This paper presents the first large-scale qualitative examination of how newsprint media framed the debate around the harms of alcohol consumption to ‘others’ during the development and passing of minimum unit pricing legislation in Scotland.

**Methods:**

Qualitative content analysis was conducted on seven UK and three Scottish national newspapers between 1 January 2005 and 30 June 2012. Relevant articles were identified using the electronic databases *Nexis UK* and *Newsbank*. A total of 403 articles focused on the harms of alcohol consumption to ‘others’ and were eligible for detailed coding and analysis.

**Results:**

Alcohol harms to wider society and communities were identified as being a worsening issue increasingly affecting everyone through shared economic costs, social disorder, crime and violence. The availability of cheap alcohol was blamed, alongside a minority of ‘problem’ youth binge drinkers. The harm caused to families was less widely reported.

**Conclusions:**

If news reporting encourages the public to perceive the harms caused by alcohol to wider society as having reached crisis point, a population-based intervention may be deemed necessary and acceptable. However, the current focus in news reports on youth binge drinkers may be masking the wider issue of overconsumption across the broader population.

## Introduction

On 24 May 2012, the Scottish Government passed legislation introducing minimum unit pricing (MUP) of alcohol (at a level of 50 pence per unit) as a targeted means of reducing the cheapest beverages thought to be responsible for causing most harm. Excessive alcohol consumption is associated with a multitude of health problems for the drinker, including increased risk of liver disease, heart disease, teenage pregnancy, sexually transmitted infections and accidental injuries 1–5.

Health problems affecting individual drinkers constitute only one dimension of the detrimental impacts of harmful drinking. A broad range of harms arising from alcohol misuse can impact upon others at a societal, community and family level. Broader societal impacts can operate through a number of mechanisms, including reduced economic activity and increased economic costs arising from health-care, policing and prison provision 1,6. Communities can be particularly adversely affected by problems associated with intoxication, violence, hooliganism and drink-driving 7–9. At the family level, problematic alcohol consumption is associated with domestic abuse, financial difficulties and poor parenting 7,10,11. This wide range of broader harms has resulted in alcohol being deemed the most harmful substance in the United Kingdom 12. Concern about alcohol-related harm is not new. The ‘gin craze’ of the mid-18th century created what Nicholls 13 described as ‘the first modern moral panic’ (p. 128), while legislation on gin production and the temperance movement highlight steps towards controlling alcohol consumption. More recently, ‘lager louts’ in the 1980s and ‘binge drinking’ and ‘ladettes’ in the 1990s and 2000s have been prominent in policy and media debates 14,15. It is perhaps unsurprising, therefore, that MUP—the newest attempt to tackle the perceived alcohol problem—has attracted widespread media coverage.

Media coverage is known to not only influence public acceptability in the lead-up to new public health interventions 16,17, but also to shape legislative priorities in the first place 18–21. The media play a key role in setting the public health news agenda, shaping public perceptions by choosing what news to report and how to report it 22. The media's influence in shaping public understandings, beliefs and behaviours on issues has encouraged its use as a tool to provide health information to the population 20. The media therefore inform the public about health issues and threats—acting as a link between them, policymakers and politicians 20,23, either educating about alcohol or normalizing over-drinking. In this respect, Nicholls 24 suggests that the media play a role ‘in articulating shared cultural values around alcohol’ (p. 200). However, selective exposure theory suggests that people choose media sources reflecting their point of view, therefore limiting the effect of the media on audience opinion. Slater 25 suggests a ‘reinforcing spirals’ approach in which ‘media selectivity and media effects form a reciprocal mutually influencing process’ (p. 283)—individuals choose media reflecting their opinions which consequently reinforce them; they then continue to select media confirming these ideas 26.

Studies examining the mass media representations of alcohol have tended to focus on alcohol advertising and television programmes and their potential impact upon public consumption 27. Hansen & Gunter 27 identified ‘a gap in the literature on media and alcohol consumption that specifically focuses on the role that news coverage can play’ (p. 154). Furthermore, Laslett *et al*. 7 suggest there has been a general neglect of research into harms to others and alcohol.

Here we present the first in-depth analysis of how the harms of alcohol are presented in UK newspapers within the context of the development and passing of MUP legislation by the Scottish Parliament. At the time of writing, MUP faces a legal challenge (instigated by the Scotch Whisky Association) and its implementation has been delayed 28. We anticipate that this study will provide valuable insights into the media's role in shaping the policy debate around the harms to ‘others’ of alcohol consumption, and in supporting the efforts of policy advocates seeking to engage with the media.

## Method

We selected seven UK and three Scottish national newspapers (including their Sunday counterparts) with high circulation figures, and a range of readership profiles representing three genres: serious, mid-market tabloids and tabloids. This typology has been used in other print media analyses to select a broad sample of newspapers with various readership profiles 17,29. See Table 1 for the newspapers included in this study.

**Table 1:** Summary of articles (*n* = 403)

Genre	Title	Total articles	
		n	%
Serious	*Guardian* and *Observer*	27	6.7
	*Daily Telegraph* and *Sunday Telegraph*	24	5.9
	*Independent* and *Independent on Sunday*	11	2.7
	*Herald* and *Sunday Herald*	94	23.3
	*Scotsman* and *Scotland on Sunday*	80	19.9
Subtotal		236	58.6
Tabloid	*Mirror* and *Sunday Mirror*	10	2.5
	*Sun* and *News of the World*	51	12.7
Subtotal		61	15.1
Mid-market	*Daily Mail* and *Mail on Sunday*	20	4.9
	*Express* and *Sunday Express*	44	10.9
	*Daily Record* and *Sunday Mail*	42	10.4
Subtotal		106	26.3
Total		403	100

Our search period was from 1 January 2005 to 30 June 2012. We selected this time-frame to encompass a period beginning 2 years before MUP was first proposed in Scotland, and ending following the passing of the legislation by the Scottish Parliament in June 2012. Relevant articles were identified using the electronic databases *Nexis UK* and *Newsbank*, adopting the search terms ‘alcohol’ and/or ‘pricing’. This search identified 1649 articles, which were exported, printed and scrutinized (C.P., K.W.) to establish whether or not it made reference to the rationale for MUP as a means to stem the alcohol problem. After excluding duplicate articles and letters 901 articles were eligible for coding, of which 403 articles discussing the Scottish Government's MUP policy were included in this analysis as a key focus of the article was the harms of alcohol consumption to ‘others’.

To develop a coding frame, a random selection of 100 articles were read to identify key themes around alcohol and create thematic categories in the initial coding frame. Using the principles of grounded theory, further batches of 20 articles were read and coded until no new categories emerged. At this point we assessed we had reached ‘saturation’, having identified all relevant thematic categories 30. Coding of articles was conducted over a 10-week period by three coders (K.W., S.H., C.P.) working together in close collaboration, with the first coder (K.W.) checking and validating each others' coding. Clarke & Everest 31 suggest that latent qualitative content includes the investigation of deeper and perhaps unintended themes, requiring more in-depth interpretive analytical qualities of qualitative methods to make inferences from data. All text was re-read and re-coded to discover patterns and anomalous ideas. Written summaries of thematic categories and the constant comparative method 30,32 informed the interpretation of the data across the articles to consider what the key messages were and how they were framed.

## Findings

Between 2005 and 2012 403 news articles were published in these 10 newspapers, with a key focus on the harms to ‘others’ of alcohol consumption. Of these articles, 58.56% (*n* = 236) were published in ‘serious’ newspapers, 15.14% (*n* = 61) in ‘mid-market’ and 26.3% (*n* = 106) in ‘tabloid’ newspapers (see Table 1). It is perhaps not unexpected that more than half of the articles were published by ‘serious’ newspapers, as this category includes the *Scotsman* and the *Herald*—both Scottish national newspapers and therefore more likely to report on a Scottish policy debate.

### Scale of harms

**Figure 1:**
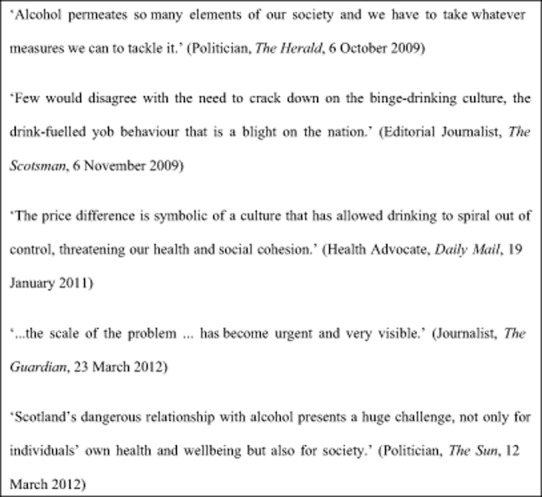
Scale of harm

A dominant theme to emerge was that the scale of harms from alcohol to people other than the drinker had reached such magnitude that urgent action was required (see Fig. 1). Articles cited evidence of spiralling economic costs, growing alcohol-related crime and violence and domestic breakdown to illustrate the extent to which alcohol consumption causes harm across society. This framing of harms to ‘others’ as reaching a ‘crisis’ (Editorial journalist, *Independent on Sunday*, 24 January 2010) ‘we can't afford to do nothing about’ (Academic, *Sun*, 26 September 2011) served as a justification for considering the new policy action. Few articles disputed the scale of the problem.

### Who is harming who?

**Figure 2:**
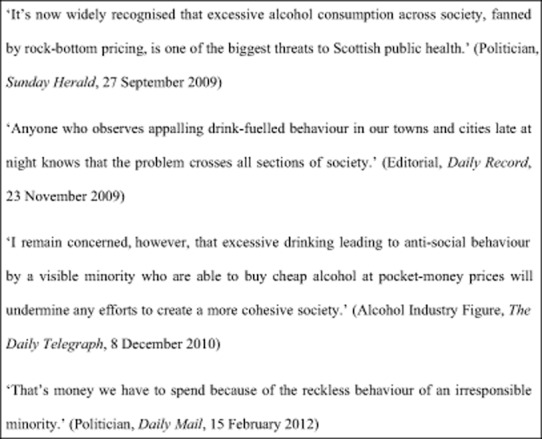
Who is harming who?

Across newspapers, alcohol consumption was widely reported as permeating every level of society, harming everybody directly or indirectly (see Fig. 2), and described as a ‘blight’ on society (Politician, *Express*, 6 June 2011). There was some divergence in whose alcohol consumption was reported to be harming ‘others’. Many articles referred to an ‘irresponsible minority’ (Politician, *Guardian*, 15 February 2012) of drinkers and also singled out young binge drinkers. Such groups were reported as becoming increasingly irresponsible in their drinking behaviours and blamed for a range of both intentional and unintentional harms to ‘others’ through their ‘alcohol-fuelled’ anti-social behaviour. Articles repeatedly mentioned ‘out-of-control’ ‘gangs of youths’ and described images of ‘… city centre streets … full of brawling, shouting, puking youngsters …’ (Editorial Journalist, *Sunday Mirror*, 25 March 2012). A second group widely identified across the newspapers were dependent drinkers who were frequently described as ‘reckless’. Both these groups were presented as the ‘visible’ or ‘problem’ ‘minority’ largely responsible for causing harms to the ‘sensible’ ‘responsible’ ‘majority’. There was a tendency to characterize ‘high-strength, low-cost alcohol’ (Government Spokesperson, *Daily Telegraph*, 30 June 2008) and ‘cut-price booze’ (Journalist, *Mirror*, 10 November 2008) as fuelling harms to ‘others’. A less common (but nevertheless observed) theme was in relation to harmful alcohol consumption at the population level. It is of interest that while some articles referred to overconsumption across the population, direct reference to groups causing harm to ‘others’, with the exception of those mentioned above, was largely absent. One article referred to ‘middle class drinkers who binge on alcohol at home’ being ‘just as responsible as drunken youths roaming the streets’ (Religious Leader, *Mirror*, 15 June 2009). Another stated that ‘behind closed doors, the prosperous and impecunious alike are drinking too much’, costing Scotland in ‘house fires and accidents in the home as well as lost working days, disease and premature death’ (Features Journalist, *Herald*, 27 November 2009).

### The economic harms to society

Economic harms of alcohol consumption were widely reported and often described as ‘spiralling’ costs. The *Observer* reported: ‘We have a problem that's costing at least £2.25bn a year, flooding our health service, undermining our economy and filling up our jails’ (Politician, *Observer*, 7 September 2008), while the *Express* stated: ‘We cannot ignore that alcohol misuse is costing £3.56 billion a year—£900 for every adult in Scotland’ (Government Spokeswoman, *Express*, 21 August 2010). Articles often used phrases such as ‘costing us’, ‘expense to the taxpayer’ and ‘we are all paying’ to generate a sense of shared harms. For example, the *Independent* stated: ‘Unlike those individual tragedies, all of us pay for the billions squandered on the National Health Service (NHS) and police costs of dealing with alcohol abuse’ (Editorial Journalist, *Independent*, 3 July 2010), while readers of the *Sun* were told: ‘… it's costing us the taxpayers’ (Alcohol Control Advocate, *Sun*, 7 May 2008). Articles frequently specifically mentioned the growing cost to the NHS and Criminal Justice System. Another reported harm was to the country's economic productivity and potential through days lost from work. However, there was some dissent from the drinks industries, who were not convinced of the economic costs (Sunday Herald, 15 March 2009). Another article questioned the accuracy of the various figures presented, suggesting they had been ‘… plucked out of the air’ (Features Journalist, *Herald*, 16 August 2010).

### Harm from social disorder, crime and violence

Antisocial behaviour and connections between alcohol and violent crime were featured consistently across newspapers. An increase in alcohol-related crime and violence was widely reported, with cheap alcohol often cited as ‘fuelling crime’ and ‘blighting our communities’ (Politician, *Sun*, 5 March 2009). Articles also referred to a rise in drunken victims of crime, reporting that alcohol not only fuels people to commit crime, but also makes people more vulnerable to becoming victims. Statistics, police reports and research evidence were used to back up these claims; for example: ‘In 2008, nearly half of Scottish prison inmates admitted being drunk when they offended’ (Journalist, *Guardian*, 11 November 2010), or reporting: ‘67% of murderers were drunk at time of killing, 450 rapes directly attributed to alcohol in 2006, 40% of jail inmates drunk when they committed offence, and 31 000 attacks last year were linked to alcohol’ (Journalist, *Sun*, 5 March 2009). In addition to criminal incidents, news articles reported on the threat of violence and subsequent fear of crime, illustrated through discussion of ‘no-go’ areas (Journalist, *Sunday Express*, 3 April 2005) where people were ‘too scared to go’ (Journalist, *Daily Telegraph* 20 January 2010). Such areas were described as ‘battle grounds’ (Editorial Journalists, *Sun*, 3 September 2010) and places that ‘you avoid at all costs’ (Editorial Journalist, *Independent on Sunday*, 24 January 2010). The role of alcohol in fuelling violence and crime, causing harm to others, was not disputed in any of the articles.

### Harm to families: home drinking and family breakdown

Another key theme in reporting was the shift away from drinking in pubs and clubs towards greater drinking in the home, attributed to the availability of cheaper supermarket alcohol. This shift in drinking patterns was reported to parallel an increase in violence occurring within homes, with the *Scotland on Sunday* reporting that ‘[t]his backs up claims by police chiefs who have warned that anti-social drinking is now more prevalent in the home rather than in pubs’ (Journalist, 17 January 2010). A key proponent of this argument in newspapers was Stephen House—then Chief Constable of Strathclyde Police—who talked about a ‘market-driven’ change in violence, warning of ‘… an increase in “private-space violence”, with fights that previously would have taken place on the street or inside licensed premises now moving into households’ (*Herald*, 6 October 2009). While violence occurring in the home was mentioned in many articles, domestic violence within families was not typically discussed in any detail. It tended to be mentioned in lists as one of a number of other problems related to alcohol consumption.

Family breakdown and harm caused to family members by alcohol abuse were also reported, with alcohol said to be ‘wrecking families’ (Editorial, *Daily Record*, 7 March 2011) and contributing to financial hardship when money is spent maintaining an alcohol addiction at the expense of the family's wellbeing:

Just about every extended family has a problem drinker. And they say every alcoholic takes five people down with them. They cause heartache to their spouse, their parents, their siblings and (if they still have one) their employer. Then there are their children … (Features Writer, *Herald*, 2 November 2010).

The particular impact of alcohol abuse on children also featured in some articles. Living with a parent who drank excessively was reported to have a negative impact on children—physical abuse, neglect and emotional stresses were reported as regular experiences. The scale of the problem was often highlighted, for example: ‘More than 2.6 m children in the UK now live with a parent who drinks at hazardous levels’ (Journalist, *Independent on Sunday*, 18 December 2011). This harm to children also extended to some reports in articles of harm caused to unborn babies by mothers drinking during pregnancy.

## Discussion

Unsurprisingly, there has been huge media interest in reporting on the development of the Alcohol (Minimum Pricing) Bill. Our analysis of UK newspaper coverage shows that harms to ‘others’ are being presented to the public as a growing and unaffordable problem that must be tackled. Newspapers portrayed the increased availability of cheap alcohol as fuelling irresponsible consumption, leading to widespread harms. This reflects the long-established evidence base for reductions in alcohol price being associated with increased alcohol consumption and alcohol-related harms 33,34. Such framing may have moved the harms to ‘others’ from alcohol consumption ‘from the realm of fate to the realm of human agency’ (35 p. 283). A commonly reported reason for the worsening situation was the shift from drinking alcohol in licensed premises towards increased consumption in domestic settings, mirroring research by the Institute of Alcohol Studies 36 and Foster & Ferguson 37.

A prominent theme to emerge was the connection between alcohol, violence and crime, which was further linked to antisocial behaviour, and public perceptions of fear in communities and cities. However, Anderson & Baumberg 1 report that fear of drunk people in public places is less common than other less severe consequences of alcohol consumption, such as being kept awake at night. This analysis shows agreement with Nicholls' 24 content analysis of television and newspaper coverage of alcohol. Both studies highlight the prominence of violence, crime and antisocial behaviour and demonstrate that they have become dominant themes in alcohol-related news reporting. It is of interest that harms to others within the family tended to play a less prominent role in articles, potentially reflecting their perceived lower salience to the general public (by either journalists, advocates or both). This may also reflect an emphasis on the more easily calculable economic, NHS and criminal impact of alcohol's harms and difficulties in calculating the impacts of alcohol abuse on a family 38.

It is noteworthy that industry figures were largely absent in the framing of the harms to ‘others’ of alcohol, perhaps indicating their focus on discrediting the policy of MUP rather than the components of the alcohol problem. Hilton *et al*. 39 provides a more detailed examination of key-claim makers and their arguments in the MUP debate.

While many articles referred to harms arising from population consumption levels, the continued concentration on specific risk groups and a minority of problem drinkers highlights a potential difficulty for those advocating for public health interventions. The concern around the drinking behaviours of young people may reflect evidence that the harms to others from their consumption have become more apparent. In addition, young people are particularly prone to experience harms from others' consumption 40. However, focusing on these harms may reinforce an emphasis on acute intoxication, down-playing the considerable burden imposed as a result of chronic consumption across the broader population. Arguably, therefore, there is a tension apparent between these presentations. On one hand, emphasizing the behaviours of specific subgroups (typically young binge drinkers) allows a clear portrayal of overt and immediate harms to society. On the other hand, Geoffrey Rose suggested that when the risk of a health harm is broadly distributed across a population, interventions to influence the overall distribution of risk may be more effective than targeting individuals at greatest risk 41. In other words, changes in population determinants of consumption (increasing alcohol price or reducing availability) may produce greater gains than targeting drinkers at highest risk. Therefore, if the public were to view alcohol harms as arising from overconsumption across the population, population-based measures (such as MUP) may be accepted more readily and the overall benefits better appreciated.

Some limitations of this research should be noted. First, as our findings are based on newspapers, the results cannot be generalized to other types of media. It would be useful for future studies to examine other media sources. Secondly, the study did not explore audience reception, and it is therefore impossible to determine how the messages presented may have been interpreted by readers. However, the study does have a number of strengths. This is the first qualitative examination of UK newspaper representations of the MUP policy and these findings may provide timely insights about the framing of messages ahead of its implementation. Conducting latent qualitative analysis was also a strength, as it allowed more in-depth investigation of data on ‘harms’ than if manifest quantitative analysis had been used alone; a paper describing trends in media coverage and the arguments presented for and against the policy, is reported elsewhere (Patterson, under review).

This media analysis of newsprint coverage during the debate on MUP in the United Kingdom shows how the case for the policy has been framed to the public. Such framing is known to influence public awareness, attitudes and behaviours, which may promote public support for policy action on alcohol and provides a case study of how the media can play a role in the development of innovative alcohol policy. In addition, this research illustrates the potential for the media to influence and increase public support for a policy by reporting on harms to ‘others’. This may, in turn, assist in achieving widespread public acceptance following the implementation of a policy, as occurred with the positive media coverage preceding the introduction of smoke-free legislation 16. Indeed, Kitzinger 42 notes that the level of media attention relates to the prominence of issues with the public and policy makers—their interest in an issue may fluctuate in response to an increase or fall in media coverage. Thus, the more news coverage an issue receives, the more important the issue may become. Giesbrecht *et al*. 43 argue that by increasing the profile of alcohol through the frame of ‘the second-hand effects of drinking’ it will be easier to develop policy responses which take account of the ‘substantial burden of illness and other harms from alcohol use’ (p. 1324–25). Babor 33 also highlights the importance of terminology, suggesting that ‘alcohol-related collateral damage brings home the realization that in many communities, homes and families, the drinking environment has become a combat zone’ (p. 1613). Our study illustrates how news reporting can encourage greater debates about the nature of harms to ‘others’ which may help to increase public support for effective targeted population health measures. However, a continued focus upon particular ‘risk groups’ may overshadow the wider issue of overconsumption across society and consequently the need for population health measures. Therefore, attempts to redress the balance in future communications may be a useful contribution to the public debate on MUP and other alcohol policies.

### Declaration of interests

K.W., C.P., S.V.K. and S.H. are funded by the UK Medical Research Council as part of the Understandings and Uses of Public Health Research programme (MC_U130085862 and MC_UU_12017/6) at the MRC/CSO Social and Public Health Sciences Unit, University of Glasgow. S.H. and S.V.K. are both involved in planning an evaluation of the impacts of the introduction of minimum unit pricing of alcohol in Scotland. The authors declare no additional conflicts of interest.
